# Pulmonary non-tuberculous mycobacteria in colonisation and disease in The Gambia

**DOI:** 10.1038/s41598-022-22777-x

**Published:** 2022-11-14

**Authors:** Catherine Okoi, Suzanne T. Anderson, Sarah Mulwa, Archibald Worwui, Martin Antonio, Florian Gehre, Ifedayo Adetifa

**Affiliations:** 1 Medical Research Council Unit The Gambia at the London School of Hygiene and Tropical Medicine, Fajara, PO Box 273, Banjul, The Gambia; 2 National Reference Laboratory, Nigeria Centre for Disease Control and Preventiuon, Abuja, Nigeria; 3 Medical Research Council Clinical Trials Unit, University College London, London, WC1E 6BT UK; 4 Faculty of Epidemiology and Population Health, London School of Hygiene and Tropical Medicine, London, UK; 5 Faculty of Infectious and Tropical Diseases, London School of Hygiene and Tropical Medicine, London, UK; 6 Microbiology and Infection Unit, Warwick Medical School, University of Warwick, Coventry, UK; 7 Infectious Disease Epidemiology Department, Bernhard-Nocht-Institute for Tropical Medicine, Hamburg, Germany; 8 Health Department, East African Community, Arusha, Tanzania; 9 Institute for Tropical Medicine, Mycobacteriology Unit, Antwerp, Belgium; 10 Epidemiology and Demography Department, KEMRI-Wellcome Trust Research Programme, Kilifi, Kenya; 11 Department of Paediatrics and Child Health, College of Medicine, University of Lagos, Lagos, Nigeria

**Keywords:** Molecular biology, Microbiology, Infectious-disease diagnostics

## Abstract

The clinical relevance of pulmonary non-tuberculous mycobacteria (PNTM) in The Gambia is unknown. The aim of this study was to estimate the prevalence of non-tuberculous mycobacteria (NTM) in colonisation, and the burden of clinically relevant pulmonary NTM (PNTM) disease in The Gambia. This was a cross-sectional study of the prevalence of NTM in participants aged ≥ 15 years, in a nationwide tuberculosis (TB) prevalence survey between December 2011 and January 2013. We enrolled 903 participants with suspected NTM and NTM cultures were confirmed by *16S rRNA* gene sequencing analyses. We applied the American Thoracic Society/Infectious Disease Society of America (ATS/IDSA) diagnostic criteria to determine clinical relevance of NTM. A total of 575 participants had acid-fast bacilli (AFB) positive Mycobacterial Growth Indicator Tube (MGIT) cultures and 229 (39.8%) were NTM. *M. avium* complex was by far the most isolated NTM (71.0%), followed by *M. fortuitum* (9.5%) and *M. nonchromogenicum* (2.9%). Older participants (> 24 years old) were four times more likely to have NTM in their sputa. Only 20.5% (9/44) NTM cases met the ATS/IDSA criteria for NTM disease. This study provides important data on the prevalence of NTM in pulmonary samples of suspected TB cases with AFB positive cultures from a nationally representative population in The Gambia. Enhanced PNTM surveillance is recommended to better understand the contribution of NTM to pulmonary disease.

## Introduction

A very large number of potentially pathogenic and non-pathogenic mycobacterial species other than the better-known *Mycobacterium tuberculosis* complex (MTBC) and *Mycobacterium leprae* are designated NTM^1^. They are ubiquitous in nature and diverse in their virulence, pathogenicity, and growth patterns^2–4^. Over 175 species of NTM have been described and the majority are very rarely implicated in clinical disease. NTM disease in humans is typically pulmonary (80–90% of all NTM disease) but other manifestations occur such as lymphadenitis in children, cutaneous disease and other extra-pulmonary or disseminated infections^5^.

The most common group of NTM in colonisation and disease is the *Mycobacterium avium* complex (*M. intracellulare and M. avium*)^6^. The Human Immunodeficiency Virus/Acquired Immunodeficiency Syndrome (HIV/AIDS) epidemic brought about great interest in the contributions of NTM opportunistic infections to morbidity in the immunocompromised host.

Most of the clinical and epidemiological data on the contribution of NTM to the aetiology of TB-like pulmonary disease are from high-income, low-tuberculosis burden settings^5,7,8^. In contrast, misdiagnosis and inappropriate treatment of PNTM disease as active pulmonary tuberculosis (PTB) may be a common challenge in many TB endemic countries^9^, including The Gambia. Despite increasingly emerging data, the epidemiology of prevalent NTM in low and middle-income countries (LMICs), also endemic for MTBC disease, is less known because of the limited availability of diagnostic modalities. In contrast to PTB, PNTM cannot be readily diagnosed using conventional laboratory tools. The culture and molecular identification techniques required for NTM diagnosis are also not readily available in resource-poor health systems where priority is given to expanding access to diagnosis and treatment of PTB^4,10^.

The American Thoracic Society/Infectious Disease Society of America (ATS/IDSA) has developed clinical, microbiologic, and radiologic criteria for identifying clinically relevant disease when NTM are isolated from pulmonary samples^5^. However, accurate assessment of true PNTM disease prevalence in many LMICs is constrained by the difficulty in obtaining complete microbiology, radiographic and clinical data.

In a recent review, the prevalence of NTM in pulmonary samples in sub-Saharan Africa was 7.5% (95% CI 7.2–7.8%) and *M. avium* complex was the most common NTM^9^. The prevalence of *M. avium* complex was five times higher in persons previously treated for PTB and/or living with HIV, while *M. kansasii* was mostly responsible for confirmed PNTM disease. NTM colonisation in presumptive TB cases, persons previously treated for TB or with chronic lung disease, may result in over diagnosis of PTB especially when smear microscopy is the only available diagnostic modality^11–13^.

Using the platform provided by Gambia’s first nationwide TB prevalence survey^11^ we investigated the prevalence and clinical relevance of NTM in pulmonary samples from a nationally representative population sample and describe the molecular epidemiology of NTM in the Gambia.

## Results

### Participant characteristics

**Figure 1:**
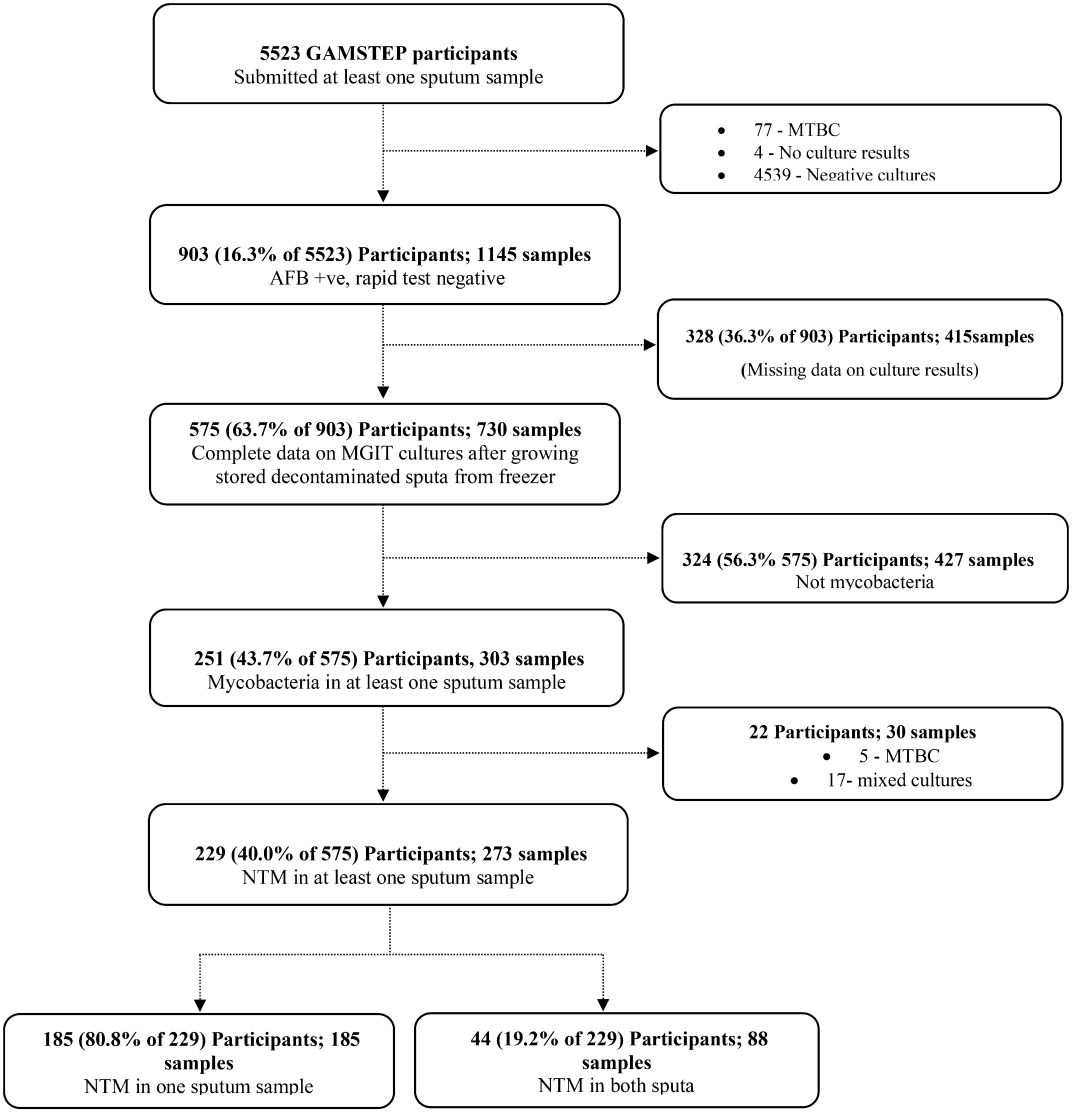
Outline of the non-tuberculous mycobacteria study in The Gambia.

Of the 903 participants with suspected NTM in the parent study, samples from 575 (63.7%) participants had positive MGIT cultures and 229 (39.8%) of these yielded NTM as shown in the CONSORT diagram (Fig. 1). The median age (Interquartile range [IQR]) of participants was 55 (40–70) years, majority (57.2%) were females and mostly (69.6%) inhabited rural parts of the country. There was no significant difference (p = 0.33) in the distribution of clinical symptoms between the overall population and those positive for NTM (see Table 1).

**Table 1:** Characteristics of the Study Population (n = 575).

Characteristics	Suspected NTM (n = 575) (%)	NTM cases (n = 229) (%)
**Gender**		
Female	329 (57.2)	141 (61.6)
**Age group (years)**		
15–24	41 (7.1)	7 (3.1)
25–34	42 (7.3)	19 (8.3)
35–44	84 (14.6)	38 (16.6)
45–54	100 (17.4)	39 (17.0)
55–64	106 (18.4)	38 (16.6)
≥ 65	202 (35.1)	88 (38.4)
**Location**		
Rural	400 (69.6)	172 (75.1)
Urban	175 (30.4)	57 (24.9)
**Clinical symptoms**		
Fever	260 (45.2)	107 (46.7)
Cough	227 (39.5)	78 (34.1)
Night sweats	64 (11.1)	29 (12.7)
Prior tuberculosis	40 (7.0)	12 (5.2)
**Ethnicity**		
Mandinka	216 (37.6)	79 (34.5)
Wolof	100 (17.4)	54 (23.6)
Fula	139 (24.2)	57 (24.9)
Others	120 (26.9)	39 (17.0)
**Occupation**		
Professional	70 (12.7)	21 (9.2)
Service worker	30 (5.2)	10 (4.4)
Agriculture	222 (38.6)	93 (40.6)
Dependant	247 (43.0)	104 (45.4)
Others	6 (1.0)	1 (0.4)
**Education**		
Literacy in English/Arabic	511 (88.9)	214 (93.5)
Primary	41 (7.1)	11 (4.8)
High school/Diploma/University	23 (4.0)	4 (1.8)
**Chest X ray**		
Normal	191 (33.2)	77 (33.6)
Abnormal	384 (67.2)	152 (66.4)
**Participant categories**		
No respiratory symptoms; normal CXR	16 (2.3)	8 (3.5)
No respiratory symptoms; abnormal CXR	266 (46.3)	103 (45.0)
Respiratory symptoms; abnormal CXR	133 (23.1)	48 (21.0)
Respiratory symptoms; normal CXR	160 (27.8)	70 (30.6)
**Prior TB**		
Yes	40 (6.96)	12 (5.25)

### Pulmonary NTM prevalence and risk factors

We estimated the prevalence of NTM in AFB positive/MPT64 negative pulmonary samples using the two statistical approaches described as shown in Table 2. The lowest prevalence estimate (25.4% [95% CI 8.8–18.4]) was seen with the most conservative model (model 2). This was the lowest limit of NTM prevalence estimate, if all unknown samples from 328 participants were negative. The prevalence estimate from model 1 (39.8% [95% CI 35.8–44.0]) in this case was the better option because it used most of the available data.

**Table 2:** Estimated prevalence of NTM in study population.

	Model 1 (N = 575)	Model 2 (N = 903)
NTM prevalence estimate (%)	39.8 (35.8–44.0)	25.4 (8.8–18.4)

Model 1 used samples from 575 participants found to have complete data on MGIT cultures after re-growing stored, decontaminated sputum samples.

Model 2 used the original number of 903 participants found in the parent study with the assumption that the negative results for 328 participants, are truly negative.

Stratification of NTM results by the different participant categories showed that the group without respiratory symptoms and normal CXR had the highest proportion of NTM positive participants compared to other groups (Table 3). It was also the smallest group, n = 16. However, these observed differences were not significant (p = 0.23).

**Table 3:** Prevalence of NTM across four participant groups in study population n = 229.

Participant categories	Total (N)	Positive n (%)	95% CI
1. No respiratory symptoms, normal CXR	16	8 (50.0)	24.7–75.3
2. No respiratory symptoms; abnormal CXR	266	103 (38.7)	32.8–44.7
3. Respiratory symptoms, abnormal CXR	133	48 (36.1)	28.0–44.9
4. Respiratory symptoms, normal CXR	160	70 (43.8)	36.0–51.8
Total	575	229 (39.8)	27.6–48.3

**Figure 2:**
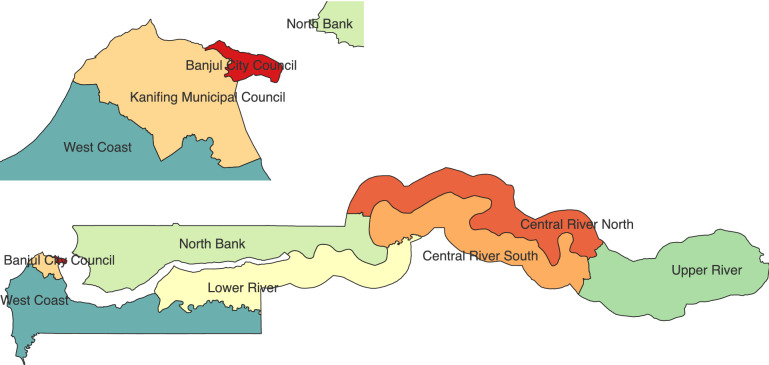
Map showing The Gambia’s six regions. Map was created using The Gambia’s administrative national and subnational boundaries shapefile downloaded from https://data.humdata.org/dataset/cod-ab-gmb?—an opensource humanitarian data exchange using QGIS version 3.24.

Univariate and multivariable regression analyses are shown in Table 4. In the adjusted regression model, the odds of a positive NTM result were approximately 4 times higher in the 25–44-year-olds and significantly higher in the > 65 age group (p = 0.003) compared to the youngest age group and females were 1.5 times more likely to be NTM positive than males (Table 4). Figure 2 displays the different geographical regions of the Gambia. Location was also significantly associated with an NTM positive culture with residents of Central River Region almost two times more likely to have NTM than Upper River Region residents, while those in Lower River were nearly half as likely as Upper River residents to have a positive NTM result.

**Table 4:** Investigating the risk factors associated with pulmonary NTM (n = 575).

Risk factor	Unadjusted odds ratio (95% CI)	*P* values	Adjusted odds ratio (95% CI)	*p* values
**Age group**				
15–24	1		1	
25–34	4.01 (1.75–9.17)	0.001	3.20 (1.41–7.25)	0.005
35–44	4.01 (1.77–9.11)	0.001	3.23 (1.36–7.68)	0.008
45–54	3.10 (1.23–7.8)	0.016	2.63 (1.01–6.87)	0.047
55–64	2.71 (1.23–5.9)	0.013	2.38(1.00–5.64)	0.050
≥ 65	3.74 (1.83–7.64)	< 0.001	3.48 (1.55–7.83)	0.003
**Sex**				
Male	1		1	
Female	1.54 (0.93–1.94)	0.112	1.50 (1.03–2.19)	0.035
**Region**				
Upper River Region	1		1	1
Central River Region	1.82 (0.06–3.12)	0.029	2.30 (1.29–4.08)	0.004
Lower River Region	0.22 (0.07–0.64)	0.006	0.23 (0.08–0.65)	0.006
Greater Banjul Region	0.58 (0.02–1.49)	0.263	0.69 (0.27–1.80)	0.457
North Bank Region	1.34 (0.78–2.30)	0.282	1.50 (0.83–2.71)	0.177
West Coast Region	0.29 (0.09–0.95)	0.040	0.35 (0.11–1.11)	0.075
**Participant categories**				
No respiratory symptoms; normal CXR	1			
No respiratory symptoms; abnormal CXR	0.63 (0.29–1.39)	0.253		
Respiratory symptoms; abnormal CXR	0.56 (0.26—1.23)	0.151		
Respiratory symptoms; normal CXR	0.78 (0.36–1.69)	0.525		
**Prior TB**				
No	1			
Yes	0.63 (0.35–5 1.14)	0.126

### Culture results and *Mycobacterium* species identification and distribution

**Figure 3:**
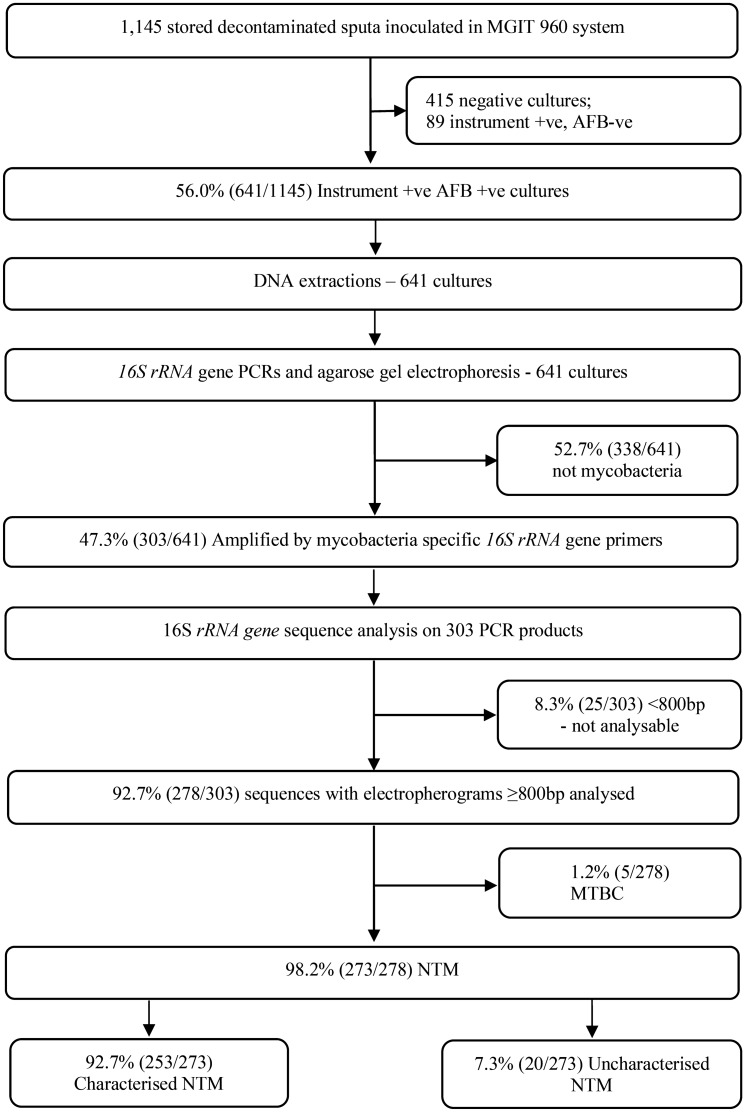
Culture results of non-tuberculous mycobacteria in eligible sputum samples at different stages of sample processing.

Figure 3 is the flowchart showing all individual samples processed for Mycobacterial speciation. There were 1145 samples from 903 participants with suspected PNTM. Sequences from 91.7% (278/303) of samples amplified as Mycobacteria were included in the final analysis. Almost all, 98.2% (273/278) of them were identified as NTM while 1.8% (5/278) cultures yielded additional MTBC. Nearly a tenth (20/273) of NTM in the study could not be characterised to species level (see Fig. 3).

### Sequence data analyses

**Figure 4:**
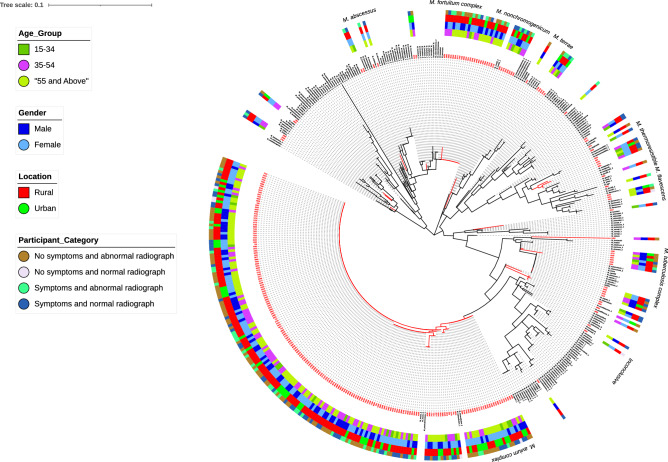
Phylogenetic tree of NTM in The Gambia based on *16S rRNA* gene sequence analysis of regions A and B of the mycobacteria gene. Red coloured fonts represent unknown sequences from the present study while fonts in black colour represent NTM and MTBC reference sequences of ATCC strains downloaded from GenBank.

The phylogenetic tree (Fig. 4) constructed using the NTM sequences obtained shows 71.1% (194/273) of all NTM sequences in this study clustered with *M. avium and M. intracellulare* (*M. avium* complex) reference strains while 10.0% (26/273) clustered with *M. fortuitum* and *M. boenickei* (*M. fortuitum* complex). Other NTM identified—*M. nonchromogenicum, M. thermoresistible*, *M. terrae* and *M. flavescens,* were 100% concordant in clustering with respective reference strains. However, 20 NTM sequences could not be identified especially as they did not cluster with any of the NTM and MTBC reference sequences. The five MTBC strains identified in the study also clustered with MTBC reference strains. Table 5 displays the Mycobacteria species identified, stratified by the different participant groupings in the present study.

**Table 5:** The distribution of non-tuberculous mycobacteria species in four participant categories.

Mycobacteria species	n (%)	No respiratory symptoms; abnormal CXR	No respiratory symptoms; normal CXR	Respiratory symptoms; abnormal CXR	Respiratory symptoms; normal CXR
*M. avium* complex	194 (71.0)	94	5	41	54
*M. fortuitum*	26 (9.5)	12	2	8	4
*M. nonchromogenicum*	8 (2.9)	1	0	3	4
*M. terrae*	5 (1.8)	1	0	2	2
*M. thermoresistible*	4 (1.5)	1	0	0	3
*M. abscessus*	2 (0.7)	0	0	1	1
*M. elephantitis*	2 (0.7)	2	0	0	0
*M. brumae-like*	2 (0.7)	0	0	0	2
*M. flavescens*	3(1.1)	0	1	2	0
*M. duvalii*	1 (0.4)	0	0	1	0
*M. goodii*	1 (0.4)	1	0	0	0
*M. cosmeticum*	1 (0.4)	0	0	1	0
*M. hodleri/isoniacini*	1 (0.4)	1	0	0	0
*M. smegmatis*	1 (0.4)	1	0	0	0
*M. triviale*	1 (0.4)	0	0	1	0
*M. komossense*	1 (0.4)	0	0	0	1
Uncharacterised NTM	20 (7.3)	7	1	3	9
Total number of cultures	273 (100)	121	9	63	80

### Prevalent pulmonary non-tuberculous mycobacteria disease

On applying the ATS/IDSA criteria to determine the clinical relevance of isolated PNTM to the 44 of 229 (19.2%) participants who grew NTM in both sputa, only 9 of 44 (20.5%) had PNTM disease, and all were caused by MAC. These nine patients met the full criteria for confirmed PNTM disease as summarised in Table 6. One participant who grew *M. fortuitum* in both sputa but did not meet clinical criteria for PNTM because they had a normal CXR. All other participants not meeting the ATS/IDSA criteria were classified as being colonised by NTM.

**Table 6:** The distribution of American Thoracic Society/Infectious Disease Society of America defined clinically relevant NTM by participant categories.

Participant category	Proportion with NTM in both samples	Proportion with NTM of same species in both samples	Meets ATS/IDSA/number
No respiratory symptoms; abnormal CXR	19/44 (43.2%)	10/44 (22.7%)	0
No respiratory symptoms; normal CXR	1/44 (2.3%)	0/44 (0.0%)	0
Respiratory symptoms; abnormal CXR	13/44 (29.5%)	9/44 (20.5%)	9
Respiratory symptoms; normal CXR	11/44 (25.0%)	8/44 (18.2%)	0
Total	44/44 (100%)	27/44 (61.4%)	9/44 (20.5%)

## Discussion

In this study we have described the epidemiology and population biology of NTM in colonisation and disease from a nationally representative population sample in a West African country.

We estimated the prevalence of NTM in pulmonary samples amongst AFB culture positives/MPT64 negative strains using two statistical approaches but settled for the complete case analysis derived estimate (39.8% [95% CI 35.8–44.0]) because it used most of the available data in the present study.

The availability of detailed clinical, radiological, and microbiologic data enabled the discrimination of pulmonary NTM carriage from disease through the application of the ATS/IDSA diagnostic criteria. When we separated the proportion of participants with NTM in both samples from those with NTM of same species in both samples, we found only 20.5% (9/44) had confirmed PNTM disease which were caused by the same NTM species—MAC, that was also the most prevalent in colonisation. This study was executed using the accepted standards for identification of NTM at every step, from culture to identification by *16S rRNA* gene analysis techniques optimised for this study^14^.

PNTM colonisation and disease rates vary globally. In North America and Europe colonisation and disease in the general population range from approximately 0.1–2/100,000 persons and 1–15/100,000 persons, respectively. However, rates are largely unknown for many countries in sub-Saharan Africa where there is a high burden of active MTBC disease^15^. In this study, the estimated prevalence of PNTM regardless of clinical relevance, was 39.8% which is higher than reported from Nigeria, Ghana, Zambia, Mozambique, Mali and Zimbabwe^16–20^. However, of all these other studies, only the Zambian and Zimbabwean studies with PNTM prevalence of 15.8% and 16.5%, respectively were also surveys of nationally representative population samples. Estimating the prevalence of PNTM in colonisation and disease is also hampered by the widely varied methods used in the limited number of published studies from sub-Saharan Africa.

The top three NTM isolated in this study (MAC, *M. fortuitum*, and *M. nonchromogenicum*) similar to reports from other African and high-income low MTBC countries where MAC was the predominant NTM species in pulmonary samples^8,15,16^. Similar to Hoefsloot et al.^21^, rapidly growing NTM (*M. fortuitum*, *M. abscessus*) accounted for a tenth of all NTM in our study. While in contrast to European and Canadian data^22^, no *M. malmoense* and *M. xenopi* were found in our study.

Distinguishing between members of the MAC family (*M. intracellulare* and *M. avium*) was incomplete in our study suggesting higher diversity in the regions A and B of the *16S rRNA* gene of Gambian MAC strains compared to those isolated elsewhere. Further evidence in support of a higher NTM diversity in our setting, was the absence of *16S rRNA* gene sequences in the National Centre for Biotechnology Information (NCBI) database for 7.3% of NTM in our study. This hypothesis requires further investigation using techniques of higher discriminatory power such as Whole Genome Sequencing.

Location was associated with increased odds of NTM in our study as has been reported by other investigators^12^. Similarly, higher risks for environmentally acquired pulmonary mycobacterial infections have also been previously reported with high soil exposure, individuals with occupational exposure to dust, and in women with high rates of biomass fuel exposure in low-income countries—all of whom are typically based in rural areas^23^. The areas of residence associated with NTM in our study are consistent with the aforementioned scenarios. Other commonly described risk factors for NTM in industrialised countries include structural lung damage (due to chronic obstructive pulmonary disease, cystic fibrosis, cumulative exposure to tobacco, prior PTB) and immunosenescence^7,8^. Increasing age and female sex were also risk factors for NTM as reported elsewhere^22^. The prevalence of ATS/IDSA confirmed PNTM was ~ 2.4 times higher in patients ≥ 50 years in one series in the US and others have found the same association for colonisation. Female sex was positively associated with NTM in this study and the literature suggests the epidemiology of NTM infection has changed over the last three decades to affect women more frequently than men. The reasons for this sex differential are unknown.

HIV is a major risk factor for PNTM and disseminated MAC infections were commonplace in high- and low-income settings before the widespread use of antiretroviral therapy^24,25^. Although we could not explore HIV as a risk factor for NTM because this data was not available, the relatively low HIV prevalence of 1.8% [1.4–2.3] in the general population at the time of the parent study suggests HIV co-infection is most likely low^13^. The TB prevalence survey was not primarily designed to study NTM which may be one reason for the low numbers of ATS/IDSA confirmed PNTM cases. The clinical relevance of NTM in younger children could not be ascertained as only participants aged 15 years or more were recruited in the parent study. Although PNTM is predominantly reported in older age groups, it is believed to affect children as well, but the epidemiology remains poorly described^17^.

WGS techniques with higher discriminatory power would have been useful for full identification of the NTM and for differentiating MAC species in this study.

In conclusion, this paper describes the contribution of NTM to colonisation and pulmonary disease in The Gambia. Only a fifth of all NTM positive participants met the rigorous ATS/IDSA diagnostic criteria confirmed disease. The high colonisation rates of NTM in pulmonary samples may contribute to the over diagnosis of PTB, especially in those with abnormal chest radiographs and where access to culture or PCR-based diagnostics do not exist as is the case in the majority of settings in The Gambia. At the time of the parent study, access to PCR diagnosis and liquid TB cultures was limited to one research laboratory in the entire country. The situation is different now with the availability of GeneXpert devices and TB culture capacity in the National Public Health Laboratory. More research and surveillance is needed to investigate the full contribution of NTM to pulmonary disease, particularly in high risk groups, and of NTM colonisation to overdiagnosis and treatment of PTB.

## Materials and methods

### Study participants and samples

Subjects were recruited as part of the Gambian national TB prevalence study, a multistage cluster survey to estimate the burden of smear positive and bacteriologically confirmed PTB, conducted between December 2011 and January 2013. During the survey, one or more sputum samples were obtained from participants with respiratory symptoms and/or abnormal chest X-ray suspected of PTB. Smear microscopy, decontamination, liquid cultures, and isolation of MTBC were performed using recommended standards and techniques as previously described^11^. The final radiological diagnosis was determined through consensus by a pulmonologist and radiologist.

From the parent survey, 903 suspected cases of NTM were identified from AFB positive cultures that tested negative for MTBC with the rapid identification assay BD MGIT™ TBc Identification (Becton, Dickinson and Company, USA). This assay tests for the MTBC-specific *Mycobacterium* Protein Target 64 (MPT64) antigen. All 903 were selected for inclusion in our study (Fig. 1). Following retrieval of stored sputa, only samples for 575 (63.7%) of 903 participants yielded AFB positive cultures, all of which were selected for analysis.

The study population was categorized into four categories: 1—no respiratory symptoms with abnormal chest X-ray (CXR); 2—no respiratory symptoms with normal CXR; 3—respiratory symptoms with abnormal CXR, and 4—respiratory symptoms with normal CXR. Participants with NTM positive samples were assessed for clinically relevant disease by application of the ATS/IDSA diagnostic criteria and classified as confirmed, probable, suspected and non-PNTM disease.

### Laboratory procedures for NTM identification

All laboratory procedures were performed at the TB Reference Laboratory, Medical Research Council Unit The Gambia at the London School of Hygiene & Tropical Medicine (MRCG at LSHTM). Sputa corresponding to AFB positive cultures and MPT64 rapid test negative in the original prevalence survey, were retrieved. Briefly, 0.5 mL of stored, decontaminated sputum samples from each suspected NTM case were cultured in Mycobacteria Growth Indicator Tubes (MGIT) supplemented with 0.8 mL of BACTEC™ MGIT™ growth supplement and BBL™ MGIT™ PANTA™ antibiotic^26^. MGIT tubes were incubated at 37 °C in the automated BACTEC MGIT 960™ system (Becton Dickinson Diagnostic Instrument Systems, Franklin Lakes, United States of America) until flagged as positive. Samples that failed to show any growth after 42 days of incubation in the machine were removed and classified as negative based on the manufacturer’s protocol. All cultures confirmed as acid-fast bacilli (AFB) with Ziehl–Neelsen (ZN) staining were classified as suspected NTM while non-AFB cultures were considered contaminants and excluded from the study.

### *16S rRNA* gene PCRs and sequencing

Mycobacterial deoxyribonucleic acid (DNA) was extracted from MGIT cultures for *16S rRNA* gene Polymerase Chain Reactions (PCR) by the boiled lysate method described by Aldous et al.^27^. Briefly, 0.5 mL of culture was centrifuged at 10,000 rpm for 15 min. The resulting pellets were resuspended in 0.1 mL sterile Tris ethylene diamine tetra acetic acid (TE) buffer^®^ (Sigma Aldrich, St. Louis, Missouri, United States) and heated to 99 °C in a heat block for 20 min followed by sonication for 15 min. Tubes were spun at 14,000 rpm for 5 min following which the supernatant was used for mycobacteria species-specific *16S rRNA* PCRs and 2.5 μL of extracted DNA was added to 22.5 μL of master mix. We used primer pairs P1 (TGCTTAACACATGCAAGTCG) and P2 (TCTCTAGACGCGTCCTGTGC) to amplify regions A and B of the mycobacteria *16S rRNA* gene. The PCR comprised a denaturation step of 5 min at 95 °C, then 45 cycles of 95 °C for 45 s, 56 °C for 45 s, 72 °C for 45 s and a final extension step at 72 °C for 10 min. Amplified DNA products were viewed on a 1% (w/v) agarose gel stained with 500 ng/μL ethidium bromide.

*16S rRNA* gene sequencing was performed on purified PCR products by the Sanger chain-termination DNA sequencing method^28^. The obtained sequences were edited and analysed in BioEdit version 7.2.5 Ibis Biosciences http://www.mbio.ncsu.edu/BioEdit/bioedit.html. Background and ambiguous non-standard nucleotides were removed and the correct DNA nucleotides inserted. Consensus sequences were computed by matching forward and reverse traces of each sample using SeqTrace 0.9.0 software^29^ http://seqtrace.googlecode.com. We filtered low quality base calls and performed end trimming. High quality finished unknown sequences were exported in Fasta format for downstream analyses. NTM and MTBC *16S rRNA* reference sequences derived from American Type Culture Collection (ATCC) isolates were downloaded from Gen Bank to illustrate the phylogenetic relationship of unknown sequences and identify them. These sequences were aligned together with unknown sequences in BioEdit software using ClustalW2 Multiple Sequence Alignment tool. A Phylogenetic tree was constructed from the aligned sequences based on regions A and B of the Mycobacteria gene using maximum likelihood algorithm. Unknown sequences were further identified based on their relatedness to NTM and MTBC reference strains on the phylogenetic tree. All sequences were made publicly available in GenBank with accession numbers KX607141.1 to KX607408.1.

### Data management and statistical analysis

Data collection and management for the parent study have been described previously^11^. All data analyses for this study were carried out with Stata version 12 (Stata Corp, College Station, TX, USA). The main outcome of the study was the prevalence of NTM amongst AFB positives cultures/MPT64 negative strains in pulmonary samples of a nationally representative population investigated for PTB in The Gambia. NTM prevalence and other categorical variables were summarised using frequency counts and proportions.

Following retrieval and re-culture, samples for 328 of the 903 originally AFB culture positives in the parent study failed to grow. To account for this disparity, NTM prevalence was estimated using two models: Model 1 in a complete case analysis used only results from the 575 participants with positive cultures after retrieval of stored sputum samples. Model 2 assumes the negative results for 328 of 903 participants, are truly negative.

Associations between NTM prevalence and categorical risk factors were examined using logistic regression models. Variables reaching statistical significance (p < 0.05) in univariate analyses were included in multivariable logistic regression models to obtain odds ratios and their 95% confidence intervals. All models adjusted for clustering as per the parent study design.

### Ethical approval

The Joint MRCG at LSHTM/Gambia Government Ethics Committee approved this study (SCC number 1371). Subjects were recruited as part of the Gambian national TB prevalence study and gave informed consent prior to enrolment. All the laboratory methods in this study were carried out in accordance with the relevant institutional guidelines and regulations.

## Supplementary Information

Supplementary Information.

## Data Availability

All relevant data are within the manuscript and its Supplementary Information file.
